# Burn-induced heterotopic ossification from incidence to therapy: key signaling pathways underlying ectopic bone formation

**DOI:** 10.1186/s11658-021-00277-6

**Published:** 2021-07-27

**Authors:** Xianglin Hu, Zhengwang Sun, Fengfeng Li, Chaoyin Jiang, Wangjun Yan, Yangbai Sun

**Affiliations:** 1 Department of Musculoskeletal Surgery, Fudan University Shanghai Cancer Center, Shanghai, 200032 China; 2 Department of Oncology, Shanghai Medical College, Fudan University, Shanghai, 200032 China; 3 Department of Orthopedic Surgery, The Second Affiliated Hospital of Guangzhou Medical University, Guangzhou, 510260 China; 4 Department of Orthopedic Surgery, Shanghai Sixth People’s Hospital, Shanghai Jiaotong University , Shanghai, 200233 China; 5 Department of Plastic and Reconstructive Surgery, Shanghai Ninth People’s Hospital, Shanghai Jiao Tong University School of Medicine, Shanghai, 200011 China

**Keywords:** Burn injury, Heterotopic ossification, Incidence, Risk factor, Signaling pathway, Mechanism

## Abstract

Burn injury is one of the potential causes of heterotopic ossification (HO), which is a rare but debilitating condition. The incidence ranges from 3.5 to 5.6 depending on body area. Burns that cover a larger percentage of the total body surface area (TBSA), require skin graft surgeries, or necessitate pulmonary intensive care are well-researched risk factors for HO. Since burns initiate such complex pathophysiological processes with a variety of molecular signal changes, it is essential to focus on HO in the specific context of burn injury to define best practices for its treatment. There are numerous key players in the pathways of burn-induced HO, including neutrophils, monocytes, transforming growth factor-β1-expressing macrophages and the adaptive immune system. The increased inflammation associated with burn injuries is also associated with pathway activation. Neurological and calcium-related contributions are also known. Endothelial-to-mesenchymal transition (EMT) and vascularization are known to play key roles in burn-induced HO, with hypoxia-inducible factor-1 (HIF-1) and vascular endothelial growth factor (VEGF) as potential initiators. Currently, non-steroidal anti-inflammatory drugs (NSAIDs) and radiotherapy are effective prophylaxes for HO. Limited joint motion, ankylosis and intolerable pain caused by burn-induced HO can be effectively tackled via surgery. Effective biomarkers for monitoring burn-induced HO occurrence and bio-prophylactic and bio-therapeutic strategies should be actively developed in the future.

## Introduction

Burn injury refers to tissue damage caused by various heat factors. These include thermal sources (fire, hot liquid and metal, and superheated steam), chemical substances (acids and alkalis), high voltages, and radiation [1–3]. The burn depth and area are the most important determining factors of burn severity [4]. Burn injuries generally occur to the skin and mucous membranes, although subcutaneous and submucosal tissues, muscles, bones, and even internal organs can be injured in severe cases [5–7]. Because the normal skin barrier function is impaired, severe burns often cause extensive tissue necrosis and fluid exudation, accompanied by shock, infection, sepsis, multiple organ dysfunction syndrome (MODS) and even death [8–10].

The pathophysiology of burns is complex, with substantial inflammatory, immune and metabolic reactions throughout their course. Patients with severe burns may experience stages of fluid exudation, acute infection, wound healing and rehabilitation [11]. Based on the latest expert opinions, wound healing is only a medium-term goal: complete recovery must address long-term complications as well as improving patient mental health and quality of life [12]. The sequelae of severe burn injury include local scarring, contracture deformity, and hypofunction of vital organs such as the heart, brain and kidneys [13–16].

Heterotopic ossification (HO) is a rare but debilitating pathological condition in which true bone tissue occurs and matures in soft tissues [17]. Unlike calcification lesions, HO leads to a complete bone microenvironment with bone tissue cells, microcirculation and neuroendocrine function [18, 19]. HO patients can experience pain and limited range of motion (ROM), which seriously impairs their daily life [20]. Burn injury is a significant source of acquired HO [21]. Burn-induced HO has been characterized in many clinical and preclinical studies and the related molecular mechanisms are gradually being elucidated.

In this review, we summarize the clinical characteristics and potential mechanisms of burn-induced HO. We also indicate the current challenges and future directions in the research and treatment of burn-induced HO.

## Clinical characteristics of burn-induced HO

### Incidence and risk factors

HO incidence after burn injury is influenced by a number of factors. Levi et al. [21] collected data on 2797 patients with burn injury from six burn centers in America and found that 98 patients  developed HO (an incidence of 3.5%). Dependent risk factors included arm burns requiring skin grafts (OR = 96.4, 95% CI 1.19–7806); burns covering more than 30% of the total body surface area (TBSA; OR = 11.5, 95% CI 6.0–21.9); multiple trips to the operating room (OR = 1.32, 95% CI 1.18–1.40); and the number of days on a ventilator (OR = 1.034, 95% CI 1.03–1.04) [21]. Similarly, Schneider et al. [22] identified that percentage TBSA and need for skin grafts on the arm, head, neck and trunk are the remarkable predictors for HO. Thefenne et al. [23] enrolled 805 patients at a burn center in France and found that 32 patients later developed HO (an incidence of 4.0%). The use of a fluidized bed (OR = 39.6, 95% CI 10.4–150.5), curare use (OR = 24.1, 95% CI 8.3–70.5), pulmonary infection (OR = 21.5, 95% CI 6.0–77.4), cutaneous infection (OR = 7.5, 95% CI 3.0–18.6), the length of stay in the intensive care unit (OR = 1.1, 95% CI 1.1–1.2), the mean total burn area (OR = 1.1, 95% CI 1.1–1.2), mean depth of the burns (OR = 1.1, 95% CI 1.1–1.2) were found to be independent risk factors for HO [23]. Orchard et al. [24] enrolled 337 patients at a burn center in Australia and found that 19 patients later developed HO (an incidence of 5.6%). A greater percentage TBSA, inhalation injury, mechanical ventilation, the number of surgical treatments, sepsis, and longer time to active movement were found to be associated with HO in that study [24].

Based on the three large burn center reports mentioned [21, 23, 24], we summarized that the incidence of HO in burn injury is about 3.5–5.6% (Table 1). Patients with burn-induced HO are mainly middle-aged people and males. The mean or median burn percentage TBSA associated with HO incidence is about 46–48.5%. The elbow is the most common site of burn-induced HO [21, 23, 24]. Larger percentage TBSA affected, burns requiring skin grafts, and burns necessitating pulmonary intensive care are the currently well-recognized risk factors for burn-induced HO. In addition, Klein et al. [25] found that a longer time to wound closure significantly increases the risk of burn-induced HO in the elbow.

**Table 1:** Incidence and risk factors for burn-induced HO (BIHO) reported in large scale burn centers within the past decade

Reference	Dataset of the study	Total simple size	Number of patients with BIHO	Incidence of BIHO (%)	Age of patients with BIHO (years)	Male (%) of patients with BIHO	Burn %TBSA	Location with frequency of BIHO	Time to incidence of BIHO	Factors associated with BIHO
Levi et al. [21]	Six burn centers in America	2797	98	3.5	41.25	81 (83.0)	47	NA	NA	1. Arm burns requiring skin grafts (OR = 96.4^a^) 2. Burn greater than 30% TBSA (OR = 11.5^a^) 3. Number of trips to operating room (OR = 1.32^a^) 4. Number of days on ventilator (OR = 1.034^a^)
Thefenne et al. [23]	Single burn center in France	805	32	4.0	47	20 (62.5)	48.5	Elbow (50%)Shoulder (20.3%)Hip (17.6%) Knee (10.8%) Wrist (1.3%)	NA	1. Use of fluidized bed (OR = 39.6^a^) 2. Curare use (OR = 24.1^a^) 3. Pulmonary infection (OR = 21.5^a^) 4. Cutaneous infection (OR = 7.5^a^) 5. Length of stay in the burns ICU (OR = 1.1^a^) 6. Mean total burn area (OR = 1.1^a^) 7. Mean depth of burns (OR = 1.1^a^)
Orchard et al. [24]	Single burn center in Australia	337	19	5.6	43	16 (84.2)	46	Elbow (89%) Knee and shoulder (less common).	Clinical: 37 (30–40) days Radiological: 49 (38–118) days	1. A greater % TBSA 2. Inhalation injury 3. Use of mechanical ventilation 4. Number of surgical procedures 5. Sepsis 6. Longer time to active movement (OR = 1.48^a^)

Age (years) of patients with BIHO was shown by median; burn % TBSA was shown by mean or median

*NA* not available

^a^Independent risk factors for BIHO that are statistically significant in multivariate analysis

### Presentation and diagnosis of burn-induced HO

Patient-reported movement restrictions and intractable pain are early signs of burn-induced HO. Patients can feel that their joints are locked or fused, with less ROM and sharp stabbing pains (nerve compression) [26]. A study from the Burn Model System National Database found that the presence of HO significantly increases the absolute loss of elbow flexion (adjusted median of 23.5°), which causes more serious elbow contracture [27]. Moreover, burn-induced HO not only causes physical limitations but also psychological burdens, such as worry and distress [26]. In turn, HO can induce recurrent non-healing ulcers in the old burn scar [28].

**Fig. 1:**
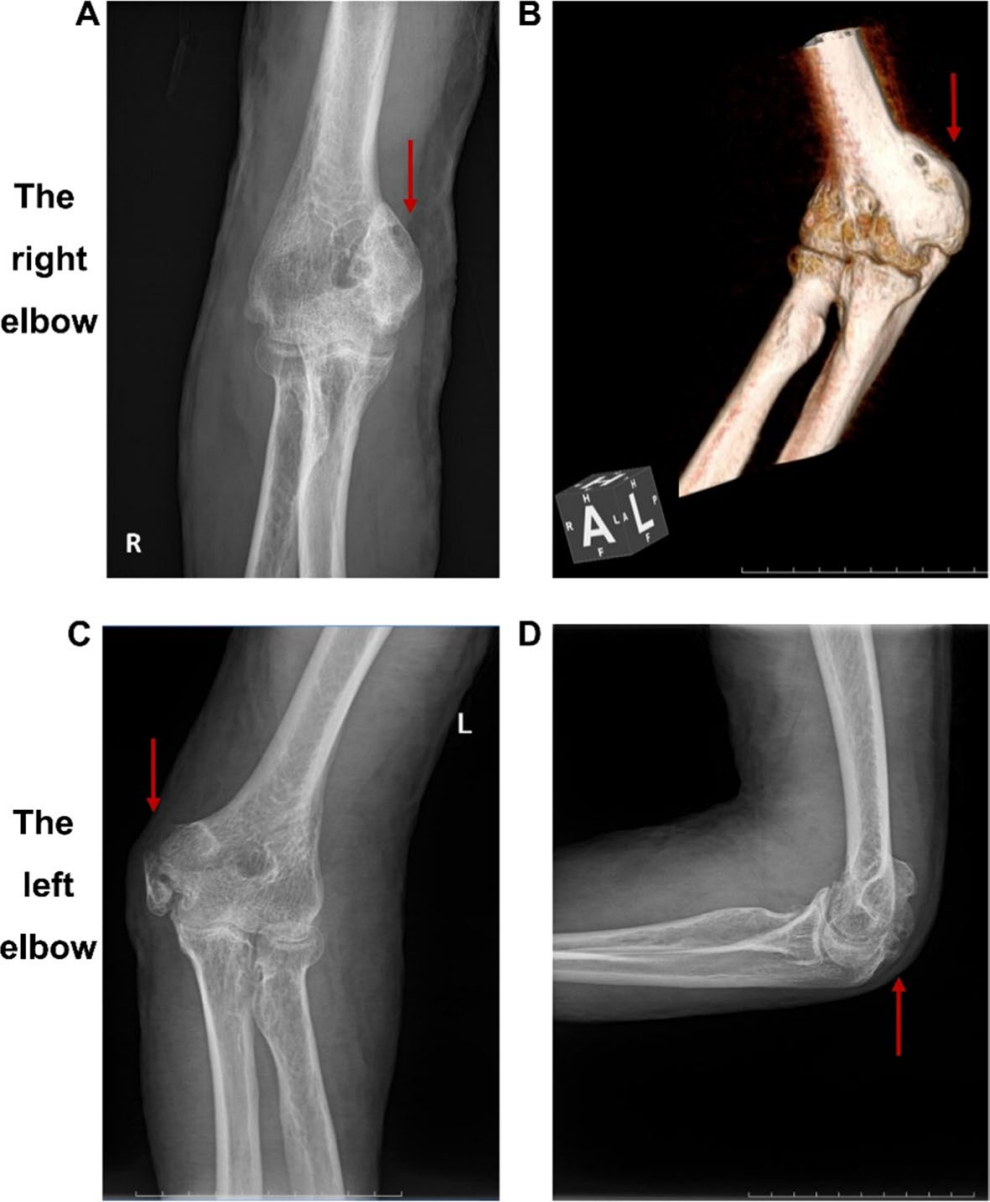
Representative imaging of burn-induced HO in the elbow. A 40-year old male patient suffered extensive thermal burn injury (80% TBSA). He complained of limitation in the range of movement of his bilateral elbow joints 3 months after the burn injury. He was diagnosed with burn-induced HO of both elbows via imaging examinations. **A** X-ray of the right elbow. **B** 3D reconstruction of CT on the right elbow. **C**, **D** X-rays of the left elbow. The red arrows indicate the HO lesions

Given their history of burns and these early manifestations, patients with HO can easily be diagnosed through imaging examinations, such as X-ray, computer tomography (CT) and magnetic resonance imaging (MRI) [29]. X-ray is a common examination for HO but only applies to mature HO lesions as it rarely identifies early-stage HO lesions. CT scans can identify tiny HO lesions early and clearly display their shape and structure [30]. MRI displays the surrounding soft tissues and can better reveal HO when used in combination with CT [31]. Although positron emission tomography-CT (PET-CT) and radionuclide bone scanning can diagnose HO with high sensitivity and specificity [32], their high costs and requirement for radioactive substances limit their use. They are not routinely recommended for HO in clinical practice. Representative imaging materials of a patient with burn-induced HO are shown in Fig. 1.

## Signaling pathways and mediators underlying burn-induced HO

**Fig. 2:**
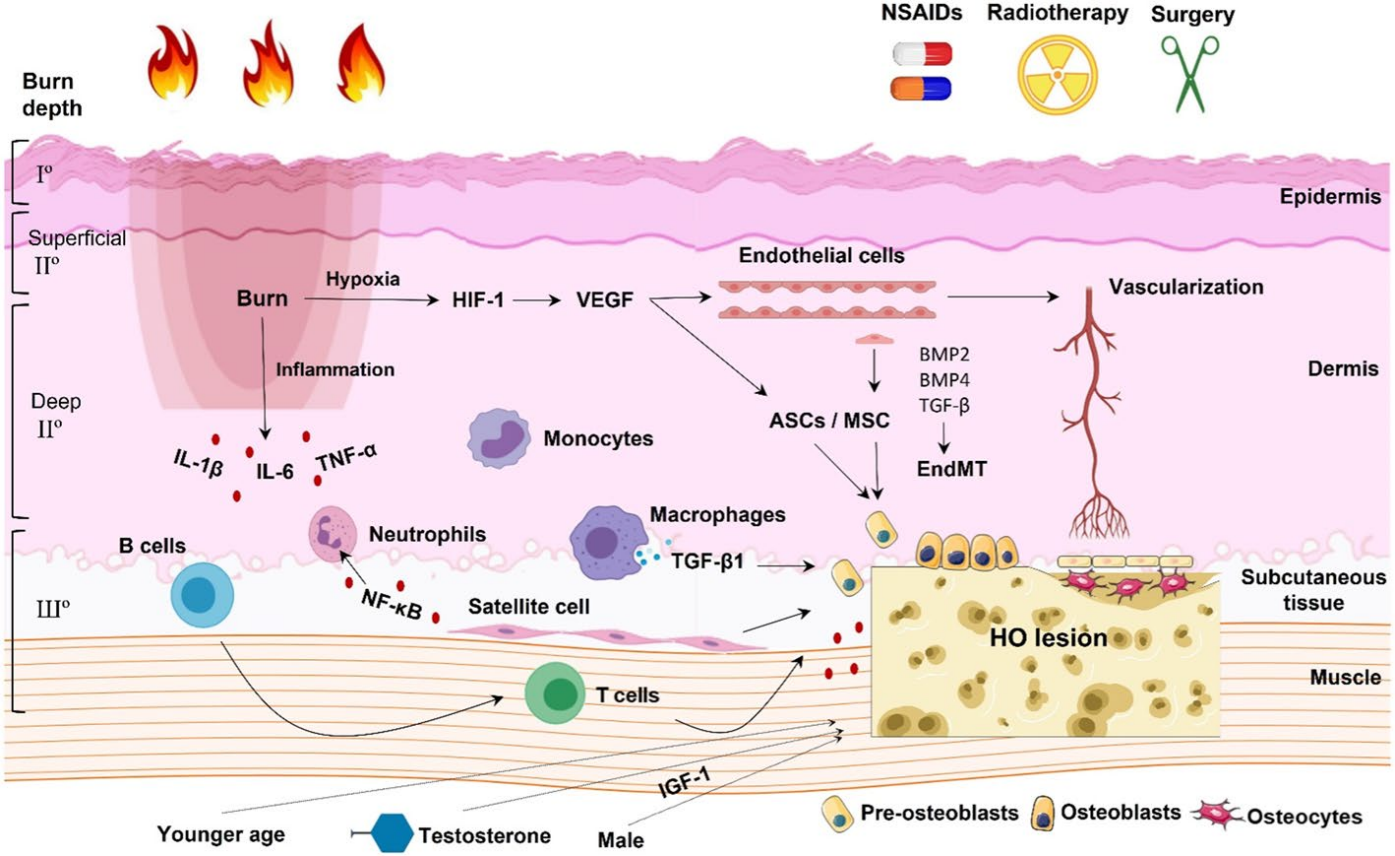
Schematic diagram showing the signaling pathways and mediators involved in burn-induced HO. Current evidence for mechanisms directly involved in burn-induced HO suggests four main pathways: vessel- and endothelial cell-based pathways; immune cell-based pathways; muscle satellite cell-based pathways; and other factors

Normal soft tissues do not have the three basic conditions required for osteogenesis, namely osteogenic precursor cells, osteogenic signal induction factors and the appropriate local microenvironment [33, 34]. It is important to investigate which cells seed in soft tissues and develop into pre-osteoblasts. The main reported potential osteogenic precursor cells (seeds) for HO are endothelial cells, muscle satellite cells, mesenchymal stem cells (MSCs), adipose-derived stem cells (ASCs), fibroblasts, tendon cells and progenitor cells [35–40]. Herein, we discuss the signaling pathways involved in burn-induced HO based on the current evidence (Fig. 2; Table 2).

**Table 2:** Key signaling pathways and potential mediators underlying burn-induced HO

Signaling pathway	Potential mediator	References
Vessel- and endothelial cell-based pathways	HIF-1, VEGF, ASCs/MSCs BMP2, BMP4, TGF-β, EMT	[43–50]
Immune cell-based pathways	TNF-α, IL-6, IL-1β, neutrophils and monocytes CXCL1, CXCL2, MCP-1, G-CSF, GM-CSF and TGF-β, macrophages T cells and B cells	[51–54]
Muscle satellite cell-based pathways	NF-κB, neutrophils, Rho signaling	[55–59]
Other factors (age, gender etc.)	Smad, NF-κB, IGF-1, testosterone	[60–62]

### Vessel- and endothelial cell-based pathways in burn-induced HO

Burn injury can cause local tissue edema and hypoxia, with a significant impact on the microvascular system. Capillary basement membrane and endothelial cells are generally impaired during a burn injury [41, 42]. In the mouse model established by Peterson et al. [43], burn injury was found to increase early vascularization and subsequent HO. It also increases tumor necrosis factor-α (TNF-α) secretion and the vascularization of ossicles [43]. Tissue hypoxia following burn injury plays a key role in the initiation of vascularization of ossicles. Hypoxia begins 48 h after the burn injury and peaks on day 3 (within the burn-healing margin), with significant expression of hypoxia-inducible factor-1 (HIF-1) and vascular endothelial growth factor (VEGF) [44, 45]. VEGF has a potent bone regeneration ability [46, 47]. Behr et al. [48] found that VEGF-A not only increases osteogenic differentiation of ASCs in vitro and in vivo, but also enhances angiogenesis of ASCs. On the other hand, using a mouse model of tenotomy with dorsal burn injury-induced HO, Agarwal et al. [49] found that VE-Cadherin-cre (a marker of endothelial cells) is positive in HO. This indicates that local and circulating endothelial cells may transform into potential osteogenic precursor cells via endothelial-to-mesenchymal transition (EMT). Bone morphogenetic protein 2 (BMP-2), BMP-4 and transforming growth factor-β (TGF-β) are considered to be the key players in EMT in HO [50].

### Immune cell-based pathways in burn-induced HO

Hyper-inflammatory levels (inflammatory cells and cytokines) and immunosuppression status are known in burn injury. The levels of serum TNF-α, interleukin 6 (IL-6), IL-1β, neutrophils and monocytes significantly increase while lymphocyte levels decrease following burn injury [51]. In a burn/tenotomy-induced HO mouse model, the site of injury shows a high local increase in the levels of monocytes and neutrophil-associated chemokines and cytokines, including CXCL1, CXCL2, monocyte chemotactic protein 1 (MCP-1), granulocyte colony-stimulating factor (G-CSF), granulocyte-macrophage colony-stimulating factor (GM-CSF) and transforming growth factor beta (TGF-β) [52]. The authors used single-cell RNA sequencing to show that the recruited monocytes and macrophages are the main culprits. They further revealed the TGF-β1-expressing macrophages drive HO formation in the burn/tenotomy model in the early stages of inflammation. Besides the predominant role of macrophages, the adaptive immune system also participates in burn-induced HO. The osteogenic capacity of MSCs decreases and HO development is attenuated without mature B- and T-lymphocytes [53]. The dysregulation of immune checkpoints on T cells and B cells might be involved in HO development [54].

### Muscle satellite cell-based pathways in burn-induced HO

Even if a cutaneous burn affects tissues isolated from skeletal muscles, it can cause myophagism, which is activity in the muscle due to neutrophils releasing nuclear factor kappa light chain enhancer of activated B cells (NF-κB). Muscle progenitor cells thus respond to cutaneous thermal injury [55]. Skeletal muscle satellite cells are a type of flat cell that is attached to the surface of muscle fibers. They have the properties of stem cells: they can proliferate and differentiate to repair muscle cells when muscle fibers are injured [56]. Human muscle satellite cells have the ability to be osteoprogenitor cells, with Rho signaling acting as the switch between myogenesis and osteogenesis [57]. Wu et al. [58] indicated that skeletal muscle satellite cells are activated after cutaneous burns in rats. They can attain significant osteogenic potential after cutaneous burns, suggesting a role in burn-induced HO [59].

### Other factors associated with the mechanisms of burn-induced HO

Peterson et al. [60] found that burn injury in young mice is associated with a more marked increase in HO development, NF-κB activation, and osteoclast activity than is seen in old mice. MSCs of young mice show more osteogenesis in vitro and higher activations of Smad and NF-κB signaling after burn injury than that found in old mice. This might answer why patients with burn-induced HO are generally young to middle-aged people with a median age of 46–48.5, as we showed above rather than old people.

Since burn-induced HO is more common in male patients (62.5–84.2%, as we showed above), a gender difference in the mechanisms of burn-induced HO was investigated [61]. In a tenotomy/burn model, MSCs from male mice showed more osteogenic gene and protein expression than those from female mice. Male mice developed 35% more HO, which was related to increased p-Smad and insulin-like growth factor 1 (IGF-1) signaling at the HO lesion [61]. Testosterone might play a role in the gender difference of burn-induced HO. Thorpe et al. [62] found that acutely burned patients who received a testosterone analog treatment presented a higher incidence of elbow HO than patients without testosterone analog treatment. In a mouse model following burn/tenotomy, testosterone analog treatment conferred a trend of developing a larger volume of HO lesions [62].

### Potential neurological and calcium-related contributions to HO following burn injury

Thermal nerve injury is characteristic of severe burn injuries [63, 64]. Substance P and calcitonin gene-related peptide (CGRP) released by the injured axon can mediate a neurogenic inflammatory reaction by recruiting neutrophils, macrophages and inflammatory cytokines [65, 66]. Substance P with crosstalk of CGRP can promote the differentiation of MSCs into osteoblasts and facilitate HO development [67, 68]. Endoneurial progenitor cells can flow via endometrial vessels to the site of HO and become an important source of osteoblasts [69].

The dysregulation of calcium metabolism following severe burn injury might also participate in HO development. Burn-induced bone resorption can release calcium into the blood [70]. Calcium and ionized calcium levels could then increase to their normal limits during the late phase of burn injury [71]. Excessive calcium may deposit and facilitate the HO lesion via the nucleotide-binding oligomerization domain-like receptor protein 3 (NLRP3) inflammasome-IL-1β pathway in macrophages [72].

## Prophylaxis and treatments of burn-induced HO

Non-steroidal anti-inflammatory drugs (NSAIDs) and radiotherapy are currently the main prophylactic strategies for HO. Surgery remains the mainstream treatment for the limited joint motion and intolerable pain caused by burn-induced HO.

### NSAIDs

As mentioned above, increased inflammatory levels play a key role in HO development. NSAIDs are cyclooxygenase (COX) inhibitors that can reduce the production of inflammatory mediator prostaglandin (PG) and bradykinin, thus exerting anti-inflammatory, analgesic and antipyretic effects [73]. NSAIDs may block chondrogenic differentiation of MSCs to inhibit bone formation [74]. A large-scale meta-analysis including 29 studies showed that both non-selective and selective NSAIDs can effectively prevent HO after total hip arthroplasty. The non-selective NSAID indomethacin and the selective NSAID celecoxib are commonly prescribed [75]. A subsequent updated Bayesian network meta-analysis also confirmed the effective role of celecoxib as a prophylaxis of HO [76]. However, it is worth noting that NSAIDs may delay bone healing [77, 78]. Thus, the dose and course of NSAID treatment should be individualized. To date, there is still no direct study exploring NSAID use in prophylaxis for burn-induced HO.

### Radiotherapy

Radiotherapy with a medium biologically effective dose from 20 to 24 Gy has proven to be an effective prophylaxis for HO after total hip arthroplasty. Preoperative and postoperative radiotherapy have similar prophylactic efficacy while multiple fractions might be more effective than single-fraction radiotherapy [79]. An in vitro experiment showed that radiotherapy can suppress the BMP2 signaling pathway in MSCs, thus interfering with BMP2-mediated osteoblastic differentiation [80]. However, it needs to be noted that radiotherapy used prophylactically for HO can be accompanied by toxic responses and an increased risk of secondary malignancy [81, 82]. The requirement for radiotherapy devices and high associated costs also limit its wide application in clinical practice. Although radiotherapy has been used in the prophylaxis of trauma-induced HO, there is still no direct evidence for its efficacy.

### Surgery of burn-induced HO

Surgery of burn-induced HO can rapidly relieve the issues of limited joint motion and pain, allowing patients to regain ability. The mean ROM significantly increased from 31° preoperatively to 99° postoperatively [83]. Passive ROM exercise and continuous physical therapy (rehabilitation) are suggested to begin on day 1 after surgery [84]. It is worth noting that HO might recur even after surgery. The re-emergence of MSCs in excision sites marked by platelet-derived growth factor receptor-α (PDGFRα) expression might be the reason for this recurrence [85]. Maender et al. [86] recommend perioperative radiotherapy to decrease HO recurrence.

## Conclusion and prospects

There are fewer direct preclinical and clinical studies of burn-induced HO than of trauma- and nerve-related HO. Here, we looked at burn-induced HO from bedside to bench and back. Since burns involve such complex pathophysiological processes with numerous molecular signals, it is urgent to elucidate the mechanisms of HO in the specific context of burn injury. How do the signaling pathways and mediators interact in burn-induced HO? What is the signaling network for burn-induced HO? Which mediators are ultimately responsible for HO following burn injury? Answering these questions will facilitate our current understanding of burn-induced HO.

In addition to clinical risk factors, effective serum biomarkers for prediction of HO occurrence after burn injury should be established. Moreover, the bio-prophylactic and bio-therapeutic strategies based on the discussed molecules and signaling pathways should be actively developed for burn-induced HO.

## Data Availability

Not applicable.

## Abbreviations

MODS: Multiple organ dysfunction syndrome
HO: Heterotopic ossification
ROM: Range(s) of motion
TBSA: Total body surface area
CT: Computed tomography
MRI: Magnetic resonance imaging
PET-CT: Positron emission tomography-CT
MSCs: Mesenchymal stem cells
ASCs: Adipose-derived stem cells
TNF-α: Tumor necrosis factor-α
HIF-1: Hypoxia-inducible factor-1
VEGF: Vascular endothelial growth factor
BMP: Bone morphogenetic protein
TGF-β: Transforming growth factor-β
EMT: Endothelial-to-mesenchymal transition
IL: Interleukin
CXCL1: Chemokine ligand 1
CXCL2: Chemokine ligand 2
MCP-1: Monocyte chemotactic protein 1
NF-κB: Nuclear factor kappa-light-chain-enhancer of activated B cells
IGF-1: Insulin-like growth factor 1
CGRP: Calcitonin gene-related peptide
NLRP3: Nucleotide-binding oligomerization domain-like receptor protein 3
NSAIDs: Non-steroidal anti-inflammatory drugs
PG: Prostaglandin
COX: Cyclooxygenase
PDGFRα: Platelet-derived growth factor receptor-α
BIHO: Burn-induced HO (used in tables only)
